# Reconciling yield gains in agronomic trials with returns under African smallholder conditions

**DOI:** 10.1038/s41598-020-71155-y

**Published:** 2020-08-31

**Authors:** Rachid Laajaj, Karen Macours, Cargele Masso, Moses Thuita, Bernard Vanlauwe

**Affiliations:** 1 Universidad de Los Andes, Calle 19A No. 1-37 Este, Edificio W, Bogota, Colombia; 2 Paris School of Economics, INRAE, 48 Boulevard Jourdan, 75014 Paris, France; 3 IITA, c/o ICIPE, P.O. Box 30772-00100, Nairobi, Kenya

**Keywords:** Environmental social sciences, Agroecology

## Abstract

Increased adoption of improved agricultural technologies is considered an essential step to address global poverty and hunger, and agronomic trials suggest intensification in developing countries could result in large yield gains. Yet the promise of new technologies does not always carry over from trials to real-life conditions, and diffusion of many technologies remains limited. We show how parcel and farmer selection, together with behavioural responses in agronomic trials, can explain why yield gain estimates from trials may differ from the yield gains of smallholders using the same inputs under real-life conditions. We provide quantitative evidence by exploiting variation in farmer selection and detailed data collection from research trials in Western Kenya on which large yield increments were observed from improved input packages for maize and soybean. After adjusting for selection, behavioural responses, and other corrections, estimates of yield gains fall to being not significantly different from zero for the input package tested on one of the crops (soybean), but remain high for the other (maize). These results suggest that testing new agricultural technologies in real-world conditions and without researcher interference early in the agricultural research and development process might help with identifying which innovations are more likely to be taken up at scale.

## Introduction

Increased agricultural intensification in developing countries is seen as a promising way to address global food needs and the yield-enhancing potential of new technologies is often a prime driver of diffusion efforts and agricultural research prioritization^1^. Evidence of high potential yields resulting from agricultural research motivates many development policy interventions aiming to increase adoption of agricultural innovations. Their limited success has triggered a large literature in economics analysing why farmers are not adopting innovations research has shown to be promising^2,3^. A growing body of randomized control trials finds low levels of adoption of new technologies^4^. Apart from market imperfections and behavioural constraints, some of the literature points to low profitability and heterogeneity to help explain low diffusion^5–9^. Others document that yield gains of new technologies, when adopted on farmers’ fields in real life conditions, do not amount to the gains that were expected based on earlier agronomic findings^10,11^. This paper unpacks some reasons for the divergences between the yield gains of improved technologies in agronomic trials compared to the ones under African smallholder farming conditions, focusing on the way researchers evaluate new technologies and the interpretation of trial results by those advising development policy makers. To do so, we build on insights from the recent literature in development economics, recognized by the 2019 Economics Nobel Prize, which has shown that seemingly promising development interventions often don’t lead to the expected outcomes, due to remaining external or internal constraints, while other solutions can be more promising than anticipated exactly because they help circumvent binding constraints. The analysis helps provide a complementary explanation for the low adoption of certain technologies. While we do not intend to say that our results establish the only reason for low adoption of agricultural innovations, we aim to contribute to the broader debate by highlighting a factor that is often ignored, and by offering a method to quantify its importance.

Crop yield gaps, the gap between potential attainable yields under high-input and optimal management and the actual yields realized under farmer conditions offer an important rational for agricultural research^12^. Agronomists use different types of on-farm trials to evaluate the promise of agricultural innovations to help close the gap, which normally follow testing in experimental stations in which yield gains are measured under controlled environments (i.e. keeping factors other than the treatment constant). Biggs (1989)’ typology of participatory on-farm research distinguishes between contractual, consultative, collaborative and collegial trials, with the level of control and participation being the highest for the researchers in the contractual trials, and the highest for the farmers in collegial trials^13^. On-farm trials designed and managed by researchers are considered best suited for evaluating bio-physical performance, as external factors can be controlled for in order to isolate the impacts of the treatment^14,15^. But calculations of the differences in yields between treatment and control plots then also explicitly factor out adjustments that could reflect real-life behavioural responses. By contrast, researcher-designed but farmer-managed trials offer the advantage of incorporating farmers’ behavioural response and thus to better reflect smallholder farming conditions^16^.

We reviewed recently published work and find that the designs of on-farm trials in published research often sit somewhere between these models, with researchers providing guidance and oversight to the management, even when the latter was done by the farmers (Supplementary Table S1 online). Also, in the majority of reviewed cases, trials were conducted with selected farmers or plots that may differ substantially from the conditions of most smallholders. While in theory, farmer-managed trials are meant to follow researcher-managed trials, in practice recommendations for extension and smallholder farmers are often derived from studies on trials with selected farmers and relatively large input and involvement of researchers (Supplementary Table S1 online). Recommendations from such on-farm trials also influence the choice of innovations being promoted in extension programs, and in the growing development economics literature using RCTs focused on the constraints to adoption^4^, illustrating the importance for a better cross-disciplinary understanding of the differences and trade-offs involved with different types of trials.

There are multiple reasons why yield gains obtained in trials are not representative of the yield gains in real-world settings. Scientists not only supply (part of) the inputs for the trials, but also often select certain types of parcels or farmers and establish the agronomic practices to be applied. This is a sensible approach given common time and resource constraints, and given that scientific learning about the new inputs may be limited when they are tested in suboptimal conditions. A review of common practices in the literature (Supplementary Table S1 online) shows, however, that researchers’ influence on inputs and practices often go beyond those that are being explicitly tested. And even when farmers themselves are given more control^17–19^, they may strategically select parcels for trials. Depending on their objectives and risk management strategies, they could select parcels with above or below average fertility conditions, which can affect the response to the inputs tested. Moreover, farmers might adjust their practices and supply more (or less) effort on the trial plots^20,21^. For trial results to be relevant for returns in the real world, one needs to (implicitly) assume that farmers will make the same type of selection and management adjustments. The question becomes whether farmers will be able to make these optimal adjustments, given that they likely face many external (such as imperfect labour, credit or insurance markets) and internal constraints (including learning difficulties and behavioural biases).

Some of these issues have been recognized before, though mostly in a qualitative way^22,23^ or by studying the heterogeneity in response through ex-post correlation analysis^24^. The increase in labour costs that can come with the adoption of new technologies or practices has also been recognized as a potential reason for lower than expected diffusion of new technology packages^9,25^. To account for these factors, guidelines for agronomic trials rely on rules of thumb considering, for instance, that attainable yield is 5–30% lower than potential yield; or that technologies need to have at least a 100% rate of return (2 to 1 benefit–cost ratio) to be considered for adoption^12,26^. We build on this literature, and follow^26^ in focussing not just on yield, but also on profitability. But we go beyond the rule-of-thumb adjustments, as the implicit assumption underlying them is that any selection or behavioural adjustments would lead to an overestimate of yield increments in agronomic trials by a fixed proportion. The results from a set of trials in Kenya suggest, however, that selection and behavioural responses can lead to both over- and underestimates, as there can be both complementarity and substitutability between farmer and parcel characteristics and management practices. Hence the required adjustment may vary: It can be larger than the typical rule-of-thumb adjustment for some technologies but have the opposite sign in others. Rule-of-thumb adjustments therefore can, in some cases lead to the inclusion of innovations that are not worth adopting for a majority of farmers, and in other cases lead to the exclusion of innovations that are in fact profitable for a large share of farmers.

Selection concerns can be amplified in multiple season trials when farmers’ decision to drop out the trials is related to their performance in the trials. And even when just focusing on a single season, plots that completely failed because of management mistakes, lack of effort or animal damage are sometimes omitted from the analysis^26,27^ to obtain estimates of the bio-physical yield; or adjustments are made for plant density to correct for low germination rates or subsequent damage when those are unrelated to the tested inputs^28,29^. This is consistent with the yield definitions in both FAOSTAT and the Global Yield Gap Atlas (https://www.yieldgap.org/), defining yield as quantity harvested over harvested area (rather than area planted). This approach allows scientists to ignore losses that are not directly associated with the technology package and to assess the potential from the technology package. But as such events may also occur in real-life this may move yield increments estimates further away from those relevant to the farmers, depending on the cause of such events^30^. In their adoption decisions, farmers are likely to consider the labour time and input costs on the area that is not harvested due to external events, and will also incorporate that prevention of such shocks can be prohibitively costly.

This paper contributes with a systematic analysis of the sources of the different discrepancies in yield gains between agronomic trials and smallholder conditions and by quantifying their combined effects on yield increment calculations, using evidence from a set of agricultural research trials and detailed data collection. Our review of recently published work confirms each of those sources of discrepancies may be relevant for a large share of published studies reporting results from on-farm agronomic trials. At the same time, not all of these discrepancies will be relevant for all trials—e.g. there often is no plant density/crop failure adjustment. The paper therefore aims to illustrate the potential importance of different factors, and possibly more importantly, to show that the different adjustments do not always affect estimates of the potential gains of a new technology in the same direction. Ultimately, the extent to which the different discrepancies matter in a particular setting will depend on which farm and farmer level constraints farmers can or cannot adjust endogenously if they were to use the input packages in real life. For example, households may not be able to automatically adjust to the practices or labour required for a newly available technology to deliver the promise observed in trials^11,31,32^, or they may not necessarily immediately know which of their parcels will be most responsive to the new input package.

An ideal experiment would directly compare yield increments between farmers asked to participate to agronomic trials and other farmers asked to grow the same crop and input package in real life conditions. However, the artificiality of the experimental circumstances would imply that this second group of random farmers would unavoidably deviate from real life conditions in such an experiment. I.e. the very fact of asking farmers to grow a crop and use an input package on one of their own plots makes that plot part of an experiment, likely leading to the same selection and behavioural responses we are trying to quantify. Therefore we instead use a decomposition method where we first estimate how the yield increments of the best-bet input packages in the trials are affected by soil characteristics, management and other farmer’s characteristics. We then use this estimated relationship to predict what the yield increment would be if the soil, management and farmer’s characteristics were the ones of representative farmers in real conditions rather than the conditions observed in the trials of the community selected farmers.

To illustrate and quantify the sources of the discrepancies in yield gains between agronomic trials and smallholder conditions, we use data from agronomic research trials for maize and soybean input packages with 336 farmers in 48 villages in Western Kenya. The trials were designed and implemented by researchers from IITA, a major international agricultural research centre. The trials allow comparing plots with a best-bet fertilizer package (a combination of fertilizer selected by agronomists based on prior work in the region) with control plots. Supplementary Table S2 online documents the trials factorial design and content of the input packages. The trials were managed by farmers and designed by researchers who provided the inputs, and guidance on practices, as is the case in much of other recently published research. The researchers selected participants non-randomly by asking community members to nominate five farmers, thought to be good farmers who would be interested in participating in the trials. This follows a common procedure for trials, and leads to a selected sample of farmers, as in cases where the technicians directly select farmers based on prior interactions with certain farmers (another common procedure). One of our central hypotheses is that these methods of selecting farmers tend to generate data from a group that are systematically unrepresentative of the village as a whole (as confirmed by differences in skills and socio-economic characteristics in Supplementary Table S3 online). For this reason, we added a comparable number of participants by randomly selecting from a list of all households in the village. Detailed data were collected on output, effort, practices, plot and farmer characteristics from trial and non-trial parcels through a combination of surveys and direct observations by agronomists and local contact people. The non-trial parcels were selected based on proximity to the trial plot and having a similar crop to observe management practices in real-life conditions (see “Methods”).

## Break down of the discrepancies between the two approaches

We highlight five possible discrepancies between the yield increment estimates from agronomic research trials and those that could be expected for representative farmers in real life conditions:

1. Agricultural yield calculations sometimes incorporate an adjustment for harvests that failed either partially or completely due to, for instance, low germination or adverse weather.
2. Management and effort may be different for the trial plots than on the typical plot of a representative farmer.
3. Plot characteristics may differ between the plots selected for the trials and other plots.
4. Farmers selected to participate to agronomic research may differ in other aspects (such as skills or asset ownership), which may have effects on yield increments beyond the ones previously mentioned.
5. In multi-season trials, some farmers may not continue through all seasons. When the decision to continue with the trial is related to yields in the first season(s), it could bias the remaining sample to farmers with high returns, while it may matter less if continuation is related to other factors.

**Figure 1:**
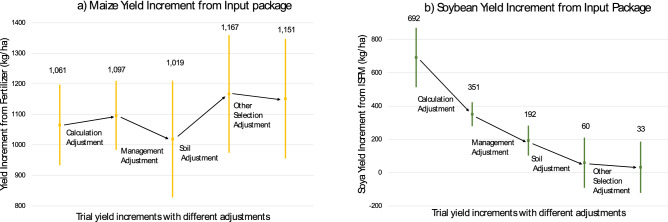
Predictions of Yield Increment Before and After Adjustments in Estimation Method. The left panel shows results for maize, the right panel results for soybean. All values are obtained from predictions of the yield increment for given calculation methods of yield increments and values of the regressors and the vertical lines represent their 90% confidence intervals. The first yield increment reported on the left side is the only one using the agronomic calculation for yield potential (correcting for plant density). The first adjustment is obtained by removing the plant density adjustment and re-inserting failed crops with zero yield, with most of the adjustment coming from the plant density adjustment (discrepancy 1). The management, soil and other selection adjustments are made by changing the value of the regressors for which the prediction is made, in order to move towards real life conditions (discrepancy 2, 3, 4). Yield increments are based on the differences in yields between treatment plots with the full input package and control plots, as explained in the methods section. Source: authors calculation, using Stata version 16 (www.stata.com) and Microsoft Excel (https://www.microsoft.com/es-co/microsoft-365/microsoft-office?rtc=1).

Figure 1 shows a series of predictions, gradually introducing adjustments for the first 4 discrepancies, starting from yield increments as calculated for agronomic trials on the left. It illustrates that the adjustments can lead to large changes in the estimation of yield increments (as is the case for soybean) but do not automatically do so (as can be seen for maize). Yield increments are calculated by subtracting yields in the control plot from the yields measured in the plot with the input package. To explain these estimates, we first focus on the explanation and estimation of each discrepancy separately, before presenting the joint analysis.

We first assess the role of management, soil and other socio-economic characteristics of the farmers involved in trials (discrepancy 2–4). These factors can differ between trials and the plots of representative farmers because of farmer selection (which can affect the three factors), because farmers tend to select different parcels (which affects soils) or because they behave differently in trial plots (which affects management). We first estimate through regressions how the management, soil and other socio-economic characteristics variables affect yield increments obtained from the best-bet input packages. As the goal is to use these estimates for predictions, we don’t estimate a production function, but rather estimate how the variation in yield increments change in relation to these factors. We then use it to predict what the yield increment would have been under real-world conditions of representative farmers (see “Methods”). These two steps allow breaking down how predictions of yield increment vary with changes in the variables that measure the three factors, as presented in Fig. 1. Each one of these three factors will affect the prediction of yield increments if the variables that measure it satisfy two conditions: (1) they matter for yield increment, i.e. they significantly predict yield increment in the trials; and (2) their values differ substantially between trial conditions and real-life smallholder conditions. We show evidence of the first condition in Supplementary Table S4 to S11 online (discussed below). Supplementary Table S3 online provides evidence on the second condition. It shows differences in management, plot characteristics, and farmers’ socio-economic backgrounds between trial plots and non-trial plots and between community selected and random farmers to illustrate the factors underlying respectively discrepancy 2, 3 and 4.

Agronomic trial designs specifically attempt to equalize management across all plots of a trial. This does not imply that management cannot affect the findings. If management practices are better, or farmers’ efforts are higher in trials than in usual farming, not only will yields in all plots be higher, but the response to the input package itself may also be higher (for instance, due to better weed control). While optimal management may make the trial most informative about the yield potential, it does not necessarily lead to estimates of yield increments that are relevant for management and effort levels that can be maintained in larger plots by smallholder farmers. To estimate the relevance of management, we use observations from the agronomist and contact person regarding the amount of weed, Striga, and the general quality of the management in both the trial plots and non-trial neighbouring parcels of the same owner. We also compare farmer’s own report about practices on the trial plots and their best managed non-trial parcel.

Management is not very different between community and randomly selected farmers, but all measures of management show significantly better management in the trials than in any other plot of the same farmers (Supplementary Table S3 online). This likely reflects the technical assistance of the agronomist, pressures to follow guidelines regarding maintenance of the trial plots, and farmer’s possible adjustment of effort to complement the best-bet input package. Supplementary Table S4 online shows regressions that predict maize yield increment and soybean yield increment as a function of the management variables observed by the agronomist and contact person. As the objective of the regressions is to obtain good predictions and to account for multicollinearity, we keep the subset of variables that maximizes the adjusted R^2^. The observed management variables alone predict 7–8% of variation in yield increment (Supplementary Table S5 online). As management practices are substantially better in trial plots, and as management matters a great deal for yield increments, we can expect that this will play an important role in the difference between calculations of yield potentials and yields in average conditions. (See further Supplementary Table S6 online for estimates with farmer reported management variables).

A similar approach helps evaluate the role of soil and plot characteristics. The soil properties (as determined from soil sample tests) do not differ significantly between community selected farmers and randomly selected ones, but survey questions do indicate that plots from community selected farmers are more likely to have had applied fertilizer in previous seasons (Supplementary Table S3 online). While we cannot directly compare soil properties of trial and non-trial plots for lack of soil samples of the later, the baseline survey data clearly indicate that parcels selected for the trial differ from other parcels in many aspects: they are more likely to be of lower quality and with Striga issues, to have been maize plots, to have had fertilizer applied in the prior season, and to be closer to the farmer’s home. Several of these characteristics correlate with soil properties, and the differences could affect yield increments in different directions. Soil properties are strong predictors of yield increment, explaining 6–7% of the variation in yield increment, while observed plot characteristics explain 3–8% (for full regressions of soil and plot characteristics see Supplementary Table S5, S7 and S8 online).

Community and randomly selected farmers also differ in other characteristics, such as skills (cognitive, non-cognitive and farming knowledge) and wealth, which could be additional drivers of yield increment estimates. The estimates suggest, however, that they are not strongly related to trial yield increments in this setting, possibly because of the relatively high degree of researcher’s control on trial management and parcel selection (Supplementary Table S9 and S10 online).

We now consider these different factors together, keeping (as before) the subset of variables that maximizes the adjusted R^2^ (Supplementary Table S11 online). The adjusted R^2^ of the full model is between 19 and 26% for yield increments—with R^2^ varying between 23 and 35%. We then use these regressions to predict yield increments under various scenarios to illustrate how differences in calculation, selection and behavioural responses lead to different estimates. We first predict yield increments under the level of management, soil and socio-economic characteristics of community selected farmers in trial conditions, the equivalent of the agronomic yield increment. The first adjustment accounts for the calculation method, as described above. We then show how yield estimates would change with management and effort that are like that applied on regular farmer’s parcels, by changing all management variables to their averages in the non-trial parcels cultivated by representative farmers. The values for representative farmers are calculated using the weighted average of the community-selected and randomly selected farmers. The weights ensure representativity of each village, putting on average 94% of the weight on the randomly selected farmers (see “Methods” section for more details). The management adjustment always lowers the yield increment, consistent with yields reacting positively to management and effort applied more intensively on the trials. We use the same approach for soil adjustment and find substantial changes as well. Notably, the effect of the soil adjustment differs between crops, with an increase for maize, but a decrease for soybean. This suggests that the input package for maize is less effective on the types of plots selected for the trials than it would be on other plots of the same farmers, leading to a potential underestimation of the real world returns. For the soybean input packages, the opposite holds. Finally, the adjustment for other socio-economic characteristics and farmers’ skills do not have any additional sizeable effect. Importantly, despite the modest R^2^ of the overall model, the confidence intervals of the predictions indicate many of the adjustments lead to significant differences.

Apart from selection and management effects, agronomic yield calculations can differ from yields obtained by farmers by including corrections for partial and complete crop failures (due to imperfect germination, animal damage, or weather hazards). Such corrections are not always done, but are often deemed useful when the factors leading to crop failure are not expected to depend on the tested technologies. Adjustment for plant density and elimination of observations with zero yield may result in a discrepancy between the yield estimated in the trial and the one obtained by farmers. To reflect the real-life yields and returns experienced by a farmer, the prior estimations include all observations whenever the plot was planted, (including plots with 0 yields because of external factors) and the yield measured as it is, without any adjustment for crop failures. We now compare these with calculations that include such corrections (discrepancy 1).

Removing adjustments for missing plants and re-introducing zero yields leads to a drop of 30% in maize yields, and 52% in soybean yield, with respectively 71 and 88% of these changes coming from the population adjustments (discrepancy 1, Supplementary Table S12 online). On average 15% stands were missing in maize trials, and 33% in soybean (compared to what it would be with full germination and all stands surviving), explaining why soybean yields drop much more with the change in calculation method. In addition, about 11% of all maize plots and 12% of soybean plots were removed because of hazard for the calculation of yield potentials but reincorporated with 0-yields in the calculation of average yields. The soybean yield increment calculation drops proportionally to the soybean yield level due to the calculation adjustment as crop failures were balanced across plots. However, the maize yield increment remains almost unchanged, because the stands population was about 91% in plots treated with the input package compared to 79% in control plots. Hence the adjustment for crop failures penalized the plots treated with fertilizer more than the control ones. Possibly fertilizer reduced Striga (a parasitic plant common in the region) and increased germination and plant survival. The adjustment takes away this effect, which may matter a lot for farmers. Overall, the first discrepancy hence has quite different implications for the input packages of maize versus soybean, as yield gain calculations for maize are largely unaffected, while they decrease a lot for soybean.

Changes in magnitude from all adjustments together are sizeable and vary between crops. All adjustments together leave the estimate of maize yield increment almost unchanged (if anything it slightly increased), while the soybean yield increment, in contrast, went from highly positive to being not significantly different from zero. The differences between the two crops are most pronounced for the calculation method, management and soil adjustments. Calculation and management adjustments lower the prediction of yield increment for soybean, while soil adjustments lead to a significant change for both maize and soybean, but in opposite directions. The adjustment for socio-economic characteristic is not significant (Supplementary Table S13 online). Importantly, as Fig. 1 shows, the qualitative conclusions hold when omitting the calculation adjustment (the first step).

The results for the yield increments, together with the cost of the input packages, allow evaluating how the adjustments affect the value-cost ratios, which is the ratio between the yield increment and the incremental cost of inputs used in the treatment plot but not in the control plot (Table 1). For the maize input package, the value-cost ratio suggests a relatively high profitability, with an average value-cost ratio around 4, which remains quite stable when predicted for representative farmers under real condition (4.2). In contrast, the average value cost ratio for the best performing soybean input package is very high before any adjustments (12), and even when accounting for the calculation method (population adjustments) suggests high profitability. However, once yield increments for representative farmers in real-life conditions are used, the value-cost ratio falls to about 0.59, indicating that it is not profitable at all.

**Table 1:** Average value-cost ratios before and after adjustments in estimation methods.

Set of variables	Maize yield increment	Soya yield increment
No adjustment, agronomic calculations	3.9	12.5
No adjustment	4.0	6.3
After all adjustments	4.2	0.6

The value-cost ratio divides the value of the additional yield by the cost of the input package, without accounting for additional labour costs (see “Methods” section for details).

**Figure 2:**
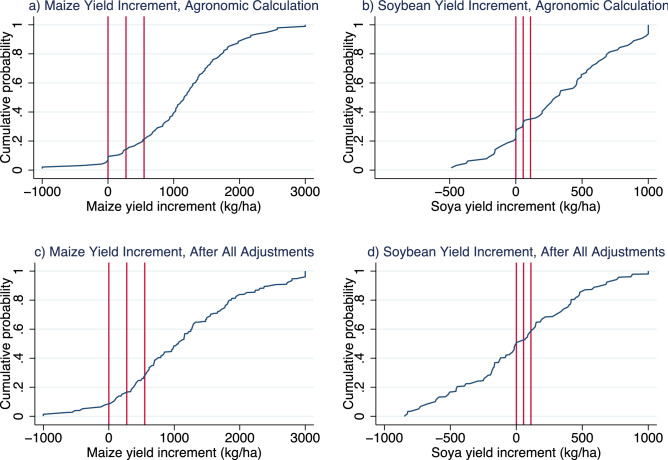
Distribution of yield increments before and after adjustments. The panels represent the cumulative distribution functions of yield increments for maize (left side) and soybean (right side). Distributions in the top row use actual yield increments, using the agronomic calculation (corrected for plant density). Distributions in the bottom row use yield increment (without the correction for plant density) after adjusting the prediction for soil characteristics, management practices and other characteristics in their own parcels, and using weights to reflect representative estimates. The first vertical line from the left indicates where the yield increment is equal to zero, the second one indicates the yield increment for which the Value-Cost ratio is equal to 1 (and thus the profit is equal to zero), and the third vertical line indicates the yield increment for which Value-Cost ratio is equal to 2 (the profit is equal to 100% of the cost). Source: authors calculation, using Stata version 16 (www.stata.com).

As average yield increments abstract from the heterogeneity in responses for different farmers and plots, Fig. 2 shows the cumulative distribution of the yield increments for both input packages, before and after the adjustments. The first vertical line shows where the yield increment turns positive, the second shows where the value-cost ratio passes 1 (corresponding to profits turning positive), and the third line shows where the value-cost ratio corresponds to the 2 to 1 ratio often used in the literature. For the maize input package after adjustments, for about 85% of farmers have a yield increment that offsets the input costs, while 79% have a predicted value-cost ratio of at least 2 to 1 with the agronomic calculations. For the soybean input package however, and even if the average value-cost ratio was much higher, when using the agronomic calculations, only 65% of farmers have a ratio above two (i.e. above the rule-of-thumb threshold). And once all the adjustments are accounted for, the input package is only found to be profitable for 47%. The relatively thick left tail of the soybean yield increment with the agronomic calculations is indeed a first indication that many farmers were not benefitting from the input package, even when the average value-cost ratio was high, and this becomes even more apparent once the different adjustments are made. Overall, the results show that the systematic ex-post adjustments commonly used in the literature are not sufficient to palliate the differences between the two approaches. That said, the results for value-cost ratios and profits come with the caveat that they do not account for labour costs, as doing so would require imposing additional assumptions that in the context of smallholder family farms can be hard to defend. Because labor costs tend to be higher in treated subplots, accounting for labour costs would reduce all value cost-ratios, but the direction of the adjustments would remain unchanged.

**Figure 3:**
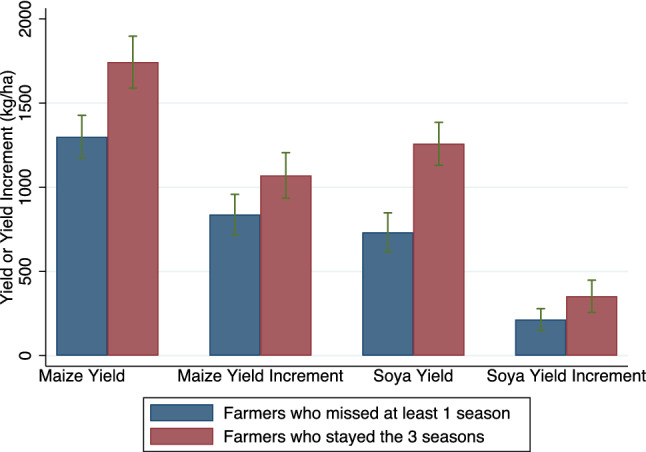
Yield and yield increment of farmers who stayed in all trials and those who missed at least one season. Average yields over 3 seasons, based on observed yield values for planted seasons, and predictions of yield for the seasons missed, as described in “Methods”. As farmers’ participation decisions likely depend on performance of all trial plots, values are averaged over all treatment and control plots. Green bars indicate the 95% confidence interval for each estimation. The proportion of farmers who participated three seasons is 33% for maize and 41% for soybean. Differences between those who missed at least one season and those that stayed 3 seasons are significant in all cases: p-values of the difference are 0.000, 0.018, 0.000 and 0.017 for maize yield, maize yield increment, soybean yield and soybean yield increment, respectively (two-sided test). Source: authors calculation, using Stata version 16 (www.stata.com) and Microsoft Excel (https://www.microsoft.com/es-co/microsoft-365/microsoft-office?rtc=1).

The results so far illustrate the possible importance of the first 4 discrepancies (and in particular the first 3) using the trial results from Western Kenya. A similar logic could be applied to other trials lasting one or multiple seasons. Discrepancy 5 applies only to multiple season trials (which include more than half of the trials in the reviewed literature). The trials in this study were conducted over three seasons, as returns to fertilizer (and in particular to inoculants) can increase over time. Multiple seasons also allow to account for differences in weather conditions, pests etc. While offering many advantages, multiple season trials can introduce attrition selection concerns when farmers’ decision to drop out the trials is related to their performance in the trials. Those who miss at least one season (67% for maize trials and 59% for soybean trials) had substantially lower maize and soybean yield and yield increments than those who completed the 3 seasons (Fig. 3, both significantly different at 5% level). Calculations keeping only the farmers that completed the 3 seasons would have led to an overestimate of 28% of the maize increment and 64% of the soybean increment. This points to the importance of aggregating results across trials and seasons, in a way that is not affected by attrition nor by seasonal variation, as we do throughout this paper (see “Methods”).

## Discussion

The results in this paper show that systematically accounting for different sources of discrepancies between agronomists’ calculations of yields from agronomic trials, and economists interests in yields in real-life conditions can result in large changes in calculations of yield increments, value-cost ratios and profits. Innovations that appear promising based on on-farm trials may be much less promising once returns are re-calculated to reflect what could be expected under real-life conditions, offering an additional explanation for the low adoption of certain innovations and for the limited impact of some innovations when adopted. The evidence further suggests that it can be hard to predict how different factors affect the estimates of yield increments, as they can lead to both under- and overestimates. This arguably calls into question common practices, which take yield as given and only account for costs when considering profits, or alternatively make rule-of-thumb corrections. Indeed, such arbitrary corrections could well lead to discarding innovations that are in fact promising for many farmers. We also show that correcting for possible selection due to endogenous selection of farmers who continue throughout multiple-season trials can be important.

For agronomists these findings imply that conclusions from on-farm trials need to be cautious, as large adjustments may be needed and extrapolation to real-world settings can be challenging, with the risk of both type 1 and type 2 errors. The promise of innovations that would perform well under average farm conditions, but not in controlled settings, could go unrecognized, while research performing well under controlled conditions may be pursued for too long. For economists interested in understanding adoption, on the other hand, the results point to the importance of understanding the differences in trial designs and the extent of control by researchers. Development economists, who often refer to such results as evidence for the promise of new technologies do not necessarily make this distinction^32–38^. The need for a management adjustment, for instance, is likely to be less important for trials that can be classified as collegial in the Biggs classification, while they are possibly more important for contractual trials^12^.

Our review of the literature points to the need for reporting guidelines in published work specifying how farmers and plots were selected, which instructions and technical assistance were given, and how crop failures and other compliance issues were accounted for. The findings also indicate that reporting both calculations of biophysical potential yield gains and yield gains to be expected under real-life conditions (as done in this paper) would facilitate use and interpretation by different stakeholders and make both agronomists and economists more aware of the implications of the different trial design choices. While this paper provides a practical example of such an approach, replication of the entire approach may not always be feasible as it requires using particular selection criteria, additional data collection and sufficiently large samples. Reporting results with and without the calculation adjustments, however, should always be feasible and is recommendable. Going beyond reporting average returns and documenting the heterogeneity in response (as discussed in^9^ and shown in Fig. 2, and as increasingly common in agronomic journals) in any given sample can also provide indications of the importance of sample selection.

The approach taken in this paper also has limitations. In particular, we need to assume that farmers’ practices on their best managed other plot help predict how they may change practices when adopting the optimal packages. As such, this approach cannot fully account for the fact that farmers may change practices further when adopting a new package in real-life conditions and may be able to purposely select the plots that are most responsive. The extent to which farmers are able to adapt plot choice and management practices to optimally match the best-bet input package is at the core of the differences between results in trial plots and possible results in real world conditions. The question becomes whether farmers can make these optimal adjustments, given that they are likely to face many external and internal constraints. In contexts where such constraints are hard to circumvent in the short-term, it can be valuable to estimate what returns would be without such full adjustments, and the paper offers a method to do so. On the other hand, in contexts where such constraints are less binding, or perhaps in the long run, when farmers may adapt to the constraints, the estimates become less relevant.

Independent of the context, and when feasible, agronomic research that accounts explicitly for plot and farmer selection and behavioural responses in the trial design could help deriving more generalizable recommendations. Some participatory research already incorporates trials designed to measure returns under farmer’s conditions, and with minimum researcher interference. Mother-baby trials^39^ in particular try reconciling the advantages of testing in controlled environments with the need to understand farmers’ constraints and preferences. The importance to understand such preferences early in the development-validation-evaluation cycle has been highlighted elsewhere^40^. The evidence in this paper shows that differences in trial results, and in their interpretation, constitutes a strong additional argument for considering such practices earlier in the innovation development process, as research orientation and diffusion efforts otherwise may well be misguided.

For those aiming to identify promising technologies for diffusion or to study diffusion of a new technology, the evidence cautions against prima facie interpretations of findings from on-farm trials. The more agronomic research incorporates calculations and designs that reflect smallholders’ conditions, the more economists and practitioners should be better able to rely on agronomic research to target research and extension efforts towards technologies with promise to increase profits in real life conditions.

## Methods

### Experimental design

The trials were conducted in five regions (Boro, Ugunja, Ukwala, Wagai and Yala) of Siaya County in western Kenya. Siaya County is located at 00°08.468′ N, 34°25.378′ E, and at an altitude of 1,336 m above sea level. The experimental sites were in lower midland 1(LM1) and lower midlands 2 (LM2) agro-ecological zones, which experience bimodal rainfall with long rains (LR) starting in March to July and short Rains (SR) starting in Late August to December^41^ and receive average annual rainfall of 1,500 mm^42^. The soils are mainly Ferralsols and Acrisols in the higher areas and Vertisols in the low areas.

Trials were conducted in 48 randomly selected villages, using stratification at the sub-county level. Half of them (randomly selected) participated in the trials in the long and short rain of 2014 and the long rain of 2015. The other half started in the short rain of 2014 and continued throughout the long and short rain of 2015. In each village 10 farmers participated in the trial. Half of them were specifically selected for participation in a community meeting. In those meetings, the researchers explained the objectives of the trials and asked the community members to nominate 5 farmers (as well as 5 potential substitutes), including two women, thought to be good farmers and interested in participating in the trials. Such non-random selection of farmers is common practice in research trials (Supplementary Table S1 online). The other half was selected randomly from the list of all the farmers in the village. All selected farmers were visited to obtain consent for the trials and identify the potential trial parcel (chosen by the farmer, conditional on fitting with some criteria for suitability to the research trials). A small number of replacements was done (but always keeping 5 selected by the community and 5 random). In each village 4 random farmers (2 random and 2 selected) were assigned to participate in the maize trial, while 3 random farmers (at least 1 random and 1 selected) were assigned to participate in the soybean trial. The other 3 farmers participated in a maize-soybean intercrop trial. During implementation, the assignment of inputs for the intercrop trial was, however, contaminated. As a result none of the plots in the intercrop trial received a best-bet input package, making the agronomic findings from that trial hard to interpret, and therefore not necessarily of interest for the decomposition proposed in this paper. Nevertheless, for completeness, results for these intercrop trials are shown in Supplementary Table S14 online.

### Researcher-designed and farmer-managed trials

The trials would qualify as researcher-designed and farmer-managed (under the supervision of the researchers). The research team had full control over the design of the trials, from the choice of inputs to spacing and other management practices. All inputs were provided by the research team, with the exception of the local maize seed tested in two out of the six plots. A researcher (local expert agronomist) was present and led planting, gapping and thinning, all fertilizer applications, and harvesting. In these activities, labor was typically provided by the farmer. Planting dates were mostly decided by the researchers to best target the onset of rains, also responding to the farmers’ feedback on beginning of rains and availability to schedule the visit for planting. The farmer was in charge of land preparation, weeding and other management, with the researchers providing guidelines on those practices. In each village, a contact person (typically one of the ten farmers) visited the trials weekly to verify that the farmers fulfilled their responsibilities. Farmers were also asked to inform the contact person in case of any pest or disease, in which case the researcher provided the required pesticide or fungicide.

### Treatment structure and application

Supplementary Table S2 online presents the full factorial designs of the multi-locational trials for maize and soybean, including details on crop varieties and quantity of inputs. The plot sizes were 4.5 × 5 m and the treatments were completely randomized between the six plots on each parcel. Plot sizes are of a similar order of magnitude as those found in other recently published work. A 1 m inter-plot spacing was planted with sweet potatoes to act as a buffer between plots to prevent inter-plot contamination. The sweet potatoes were planted at 50 cm from each plot, and border rows of the maize and soya plots were excluded for yield estimations to limit any edge effect. Hence the area harvested was 12.9 m^2^ for maize and 13.5 m^2^ for soybean. The experiments were repeated for three seasons, and plot layout and treatments were maintained for three seasons.

For the soybean trials, a soybean rhizobia inoculant was tested alone, with Minjingu hyper phosphate (0-30-0 + 38CaO) or Sympal (0:23:15 + 10CaO + 4S + 1MgO + 0.1Zn) in a full factorial design. Phosphorus rate of 30 kg P ha^−1^ was used to determine the quantity of Sympal and Minjingu hyper phosphate to be applied. On each farm only one replicate was used; hence, 6 plots were installed on each farm. Inoculation was done at planting as a seed coating using the directions for use in the respective product labels. Each plot had 6 soybean lines of 5 m in length each spaced at 5 cm from plant to plant within row and 50 cm from row to row. Inoculation was done on all the rows. Soybean variety TGx1740-2F with medium maturity (95–100 days)^43^ was used as the test crop. The spatial variability of the soybean response is studied in^44^. The soybean trials demonstrated that the combination of rhizobia inoculant and P-source led to important yield gains^44^.

The choice of inputs resulted from prior research conducted as part of the Compro project. Soybean was chosen as test crop mainly because in the prior phase of the project it had shown good response to rhizobia inoculation^45^ and was agro-ecologically suitable to the region. Kenya is an importer of soybean and multiple efforts are geared towards raising local production. In Compro I, the two rhizobia inoculants were tested and shown to be effective in increasing nodulation, nitrogen fixation and yield when inoculated on the tested soybean variety. Minjingu and Sympal were chosen based on their formulation with respect to the chemical characteristics of the soils in the test sites and results of earlier research^46^. The soils generally lack phosphorus and are acidic. A mapping study^47^ specifically identified Western Kenya as a potential K deficient area, and soil acidity has long been identified as a constraining factor in Western Kenya^48^ hence the importance of CaO. Results from soil sampling of the trial plots confirmed that more than 56.87% of soils were acidic (pH < 5.5), and more than 20.46% have low or very low K (pH < 0.160). Minjingu provides P but also reduces soil acidity because of the liming effect of the CaO, whereas Sympal also addresses potassium as well as other micronutrient deficiencies. A combination of inoculant and fertilizer was expected to perform better than the individual components as the soils are lacking both P and N, hence the combination was considered a best bet package to improve yield performance. Two different soybean inoculants were tested. Legumefix soybean from Legume technology (UK) containing *Bradyrhizobium japonicum* strain 532c^45^ and Biofix soybean from MEA Ltd (Kenya) containing *Bradyrhizobium diazoeficiens* strain USDA110^49^. Randomly selected villages were assigned to test one of the two inoculants.

For the maize trials, a package of Mavuno (10N:26P:10K: 5S: 14CaO + micronutrients) was tested at the rate of 45 kg P ha^−1^. This rate of P gave a rate of 39.6, 39.6 and 55 kg ha^−1^ of N, K and CaO) respectively. As for soybean, the use of fertilizer with K and CaO was motivated by reports of deficiency of K and soil acidity. The gains from adding K to NP for maize plots in Western Kenya have been demonstrated^50^. Other work more generally indicates that addressing limitations in secondary and micronutrients, and increasing soil carbon can improve response to fertilizer^51^. Topdressing was done with Mavuno topdressing fertilizer at the rate of 35 kg N ha^−1^. Phymix was applied at the rate of 250 kg ha^−1^ as recommended on product label. Phymix is a vermicompost with total N (0.88%), organic C (7.31%), available P (0.39%), Ca (0.29%), Mg (0.1%), K (0.22%), and a pH that is approximately neutral (6.7%). Three maize varieties were tested: local seeds, imidazolinone-resistant (IR) maize seed or a hybrid seed, randomly allocated to the 6 plots in a full factorial design. See Supplementary Table S2 online. Two different hybrid seeds were tested. KSTP94 and DH04. Randomly selected villages were assigned to test one of the two hybrid seeds.

The maize trials aimed to test some of the most viable solutions that an integrated soil fertility management combination of inputs can bring to the conditions faced by farmers in the region. A pilot phase with 60 trials in the Wagai region of Siaya county showed relatively low yields for maize because of a widespread Striga infestation problems, hence the decision to include seeds that were expected to perform better under such conditions. IR maize is marketed as a viable option to farmers with high Striga infestation problems; KSTP is a Striga tolerant variety that has high yield while DH04 is another high-yielding hybrid variety commonly grown in the area. Mavuno planting is a balanced planting fertilizer blend allowing for a split application of N as top dressing to increase N use efficiency and reduce losses at the early crop growth. The Mavuno top dressing was used for top dressing in accordance with the recommendation of split N application, as the rate used at planting cannot meet the N needs of the crop. Phymix was used as the soils are very low in organic matter and it was not possible to obtain the organic matter from sources such as compost in the quantity and consistent quality required for the trials. Being a commercial product, it ensured availability and similar quality across trials.

### Data

Soil samples were taken once before any treatments were applied and sent for analysis. Available P was determined using the Mehlich 3 method (Mehlich, 1984) while pH (H_2_O) was done as described in^52^ and exchangeable K, Ca, Mg, Total N organic carbon were determined as in^53,54^.

Detailed observations were also collected regarding crop management (quality of land preparation, absence of weed, and absence of Striga) on trial and neighbouring non-trial parcels of the participating farmers. The agronomist and contact person were asked to evaluate quality of land preparation, Striga prevalence and weed prevalence on a scale of 1 to 3. The information was filled both for the research trial area and for the area directly bordering the trial plots and cultivated by the same farmer (referred to here as the neighbouring non-trial parcel). The agronomist completed the information during 4 visits per season (planting, top dressing, biomass assessment, harvest). The local contact person visited the trials weekly and completed the form 4 times (before planting, between planting and top dressing, between one and two months, and after two months but before harvest). Agronomists and contact persons were trained for standardized application of the forms. Observations of the agronomists and contact person were averaged across all visits and all seasons, separately for trial and non-trial parcels, to create indices capturing weed absence, Striga absence, and quality of land preparation. After averaging the grades, they were rescaled so that 1 corresponds to the highest possible score and 0 to the lowest possible score (hence Striga and weed prevalence were inversed). While the amount of weed and Striga could also result from inherent plot conditions, most of those are factored out in the comparison of the trial parcel with the neighbouring parcel. We use these data sources to measure observed management.

A farmer survey was conducted prior to the first season, and again after each season. At baseline, detailed information regarding the farmer’s skills were collected, which are aggregated in separate standardized indices for cognitive, non-cognitive and technical skills, following methods explained in detail in^55^. Data on asset ownership were also collected and aggregated in a standardized wealth index using principal component analysis. The units of the skill and wealth indices correspond to standard deviation changes. We also collected data on whether the main farmer in the household (who was to be responsible for the trial) was a woman and was the household head. Data were also collected regarding plot characteristics, allowing to characterize both the parcels selected for the trials, and all other parcels cultivated with annual crops, with questions capturing subjective soil quality (on a scale from 1 to 5), inclination (scale from 1–3), area (in acres), distance to homestead (in minutes), past crops and past practices used (fertilizer, manure, erosion control)—see Supplementary Table S3 online for the specific questions. Values for non-trial parcels are averaged across parcels. Note that while self-reports on past practices suggest more past fertilizer on the trial plots of community selected farmers, the soil sampling suggested no observed differences in soil properties at the start of the trials. Possibly fertilizer was only applied in small quantities as it is commonly reported in the smallholder farming systems^56,57^.

Agronomists also collected information about pre-trial presence of Striga (this is captured by the agronomists observation of Striga infestation on the parcel, and questions to the farmer on the prevalence of Striga in the main season of 2013, and on the number of years with Striga on the parcel), which is also used to characterize the trial parcel at baseline. This information, like the soil sampling, is only available for the trial parcels.

After each trial season, parcel-level and farmer-level data were collected to characterize input use and practices on trial and non-trial parcels. Data were collected for all cereal and legume parcels cultivated by the farmer. We use this data to obtain alternative proxies of management, by constructing variables capturing whether the farmer harrowed the parcel at least twice, weeded the parcel at least twice, and did gapping or thinning to adjust for uneven germination after planting. As management practices can differ by plot, and to reflect the best management practices farmers themselves chose for their own plots, management practices for the non-trial parcels are coded based on the best practice observed among all cereal and legume parcels for each farmer. If anything, the positive differences in Supplementary Table S3 online hence should underestimate differences between trial and non-trial parcels. By using the values of the best management plot (rather than the average for mono-cropped maize and/or (soy)bean plots—which are lower), we implicitly assume that farmers would adjust practices upwards to the best they currently do on any plot. This may still, however, underestimate the behavioural response if, when adopting the best-bet package, they would be able to overcome internal or external constraints and further improve practices. In that case, the magnitude of the behavioural adjustment would be smaller, but the direction would stay the same (as long as there is no complete adjustment).

Harvest quantities, collected in each season through crop cuts, are used to calculate agricultural production on each of the 6 trial plots for each farmer. Harvesting for both soybean and maize was done at physiological maturity. Plant populations, effective area, total fresh weight before taking sub-samples were recorded. Maize grain production was determined by weighing the fresh weight of all the cobs in the inner four rows of the plot (effective area). Sample fresh and dry weights of cobs were taken and then shelling was done. This was then used to calculate grain yield per effective area and then extrapolated to hectare basis. Soybean grain production was determined by threshing grain from the effective area and determining the weight. A sample was taken, and its weight determined before drying. Thereafter these dry weights were used to calculate yield per plot and extrapolated to hectare basis. For both maize and soybean, plant density adjustments were made by multiplying the yield by the expected plant population (based on spacing at planting) with the effective plant population (number of stands in the plot before harvesting). Plots without production are omitted for the agronomic calculations.

Yield increments for maize are calculated using three pairs of treatment–control plots: T2–T1, i.e. subtracting yields in the plot with local seeds and no fertilizer (T1) from yields in the plot with local seeds and the full fertilizer package (Mavuno, planting and top dressing, and Phymix). Similarly, calculations are done for hybrid seed plots with and without fertilizer with (T4–T3), and for IR seeds plots with and without fertilizer (T6–T5). See Supplementary Table S2 and S15 online.

Yield increments for soybean are calculated using two pairs of treatment–control plots: (a) by subtracting yields in the control plot from yields in plots containing a soybean inoculant and Sympal (T6); and (b) by subtracting yields in the control plot from yields in plots containing a soybean inoculant and Minjingu (T5).

We do not use yield of the other soybean plots in the main analysis, as they contain only one of the fertilizer types and hence don’t reflect a best-bet input package. These subplots were included in the experiment as part of the factorial design of the agronomical trial, analysed in^44^. For completeness the decomposition results for the paired comparison of each of these subplots with the control plot is included in Supplementary Table S14 online, showing that the different adjustments broadly lead to qualitatively similar changes for different subplot pair comparisons.

Related, to enhance comparability, we focus the analysis on yield increments obtained from the fertilizer packages for both maize and soybean. Although the maize trials also allow comparing yield estimates of different crop varieties, we do not analyze these separately, because the differences between plots with different seeds are relatively small, which limits the statistical power of any comparison between treatment and control plots. This is consistent with earlier findings showing that IR varieties are not necessarily doing better than local varieties in absence of Striga and (ii) that the chemical that is coating the maize seed is often lost quite rapidly, thus not allowing the expression of the IR properties on maize yield^58,59^.

Supplementary Table S14 online also reports results of the intercrop trials, which were meant to follow the same factorial design and input mix for intercropped soybeans as the soybean trials. However, input application was contaminated, making the agronomic results hard to interpret and therefore of less interest for the decomposition (as illustrated by low agronomical yield increments). Decomposition results are nevertheless presented here for completeness. They show a broadly similar pattern as for the soybean trials.

### Statistical analysis

Trials were repeated for 3 consecutive seasons, to gain power and avoid results being excessively subject to weather variations in a single season. The yield increments are obtained by taking the difference between the yields in the paired treated (with the input package) and control plots. Supplementary Table S15 online displays the yield by subplot averaged over the 3 seasons, for all farmers together and separately for community selected and randomly selected farmers. When data are missing for a given season because the trial was not planted (typically because of refusal from the farmer to continue the experimentation in his/her field), we predict the value of the missing season before averaging the three seasons. The prediction uses the average value of the yield increments during the seasons for which observations for that plot are available and adjusts it for the seasonal variation. Hence to calculate the yield of a plot planted in the first and second seasons but not in the third season, we first calculate the difference in yield between the third season and the average of the first two seasons for the subsample of farmers who planted the trial in the three seasons. Then, we add this difference to the average yield from the first and second season of this plot to impute yield for that plot in the third season. This way of imputing missing yields accounts for how well the farmer’s trial is doing compared to other trials cultivated in the same seasons, and accounts for how much higher or lower yields were in the missing season(s) compared to the season(s) available.

### Calculation of adjustments in yield increments

For the yield increment predictions underlying Fig. 1, we regressed the yields increments based on all the paired best-bet treatment–control plot pairs (i.e. T6–T5; T4–T3 and T2–T1 for maize; and T6–T1 and T4–T1 for soybean) on all possible covariates listed in Supplementary Table S3 online the variable indicating whether farmer was selected by the community, and binary variables to control for constant differences between pairs. We then restricted the predictor set by conducting a stepwise selection of variables with backward elimination and using the adjusted R^2^ as information criteria. Supplementary Table S11 online shows the regressions keeping only the variables that survived the stepwise selection. We use the point estimates of that regression to obtain predictions of yield increments for different values of the regressors. As there are multiple plot-pairs per household in each regression, standard errors are clustered at the household level in the main specification. Column 3 and 4 show alternative estimates, with standard errors clustered at the village level. Comparing the results with those of column 1 and 2 shows that results are robust to this alternative specification.

The first two predictions of Fig. 1 use the values of management, soil and farmers’ characteristics of the community selected farmers as observed in the trials. The calculation adjustment uses the same value of regressors, but changes the outcome variable of the prediction by taking away the plant density adjustment and re-inserting observations with failed crops (with zero yield rather than missing). Supplementary Table S12 online shows adjustment for plant density and crop failures separately, and shows that the plant density adjustment accounts for most of the changes, as discussed above. For crop failure, farmers could not only indicate the specific reason in about half of the cases, with animal damage (32%), lack of germination (10%) and weather (9%) being the most common reasons (in LR 15). For the other half of cases, farmers either explicitly indicated they didn’t know the reason for crop failure, or indicated symptoms (e.g. crop being yellow) rather than underlying reasons. As the data hence does not include comprehensive information on the causes of crop failures all are adjusted for equally. However, given the nature of the events causing crop failure, the frequency of these events over 3 years and across 48 randomly-selected villages should give a good estimate of the expected frequency in the region of study, and hence are taken to be reflective of probabilities of such events occurring in real life conditions.

All the other adjustments illustrated in Fig. 1 reflect the changes in yield increment predictions that result from changing the values of the covariates and recalculating the yield predictions with these different values of covariates using the point estimates from Supplementary Table S11 online. The differences in covariates underlying these estimations can be found in Supplementary Table S3 online.

Specifically, the management adjustment changes the value of all management variables from the measure of management of community-selected farmers in trials to the values of management of representative farmers in their own parcels. The average value for representative farmers is obtained by using the weighted average of randomly selected farmers and community-selected farmers. For a village of size n, community-selected farmers are assigned a weight of 5/n, and farmers randomly drawn from the rest of the village are assigned a weight of (n-5)/n (i.e. the share of randomly selected farmers over the total). As average village size is 85 (ranging from 36 to 160), the value of the representative farmer is close to that of the randomly selected farmers. This adjustment can be interpreted as the effect of changing the level of management from the one in trial to the average level of management in real-life conditions.

Similarly, the soil adjustment replaces plot characteristics (according to survey answers) by the average ones of representative farmers in their own parcels and replaces the values of the soil property variables for community selected farmers by the average ones for representative farmers. But as information from soil sampling and information obtained during identification of the trial parcels is not available for non-trial parcels, we are not able to account for differences between trial and non-trial parcels of representative farmers that are not captured by the observed plot characteristics. As expected, however, several of the observed plot characteristics are strongly correlated with soil properties. For example, quality of the plot according to the farmer’s assessment, an indicator variable for prior use of manure in the parcel and an indicator variable for prior use of chemical fertilizer are significantly correlated with respectively 7, 7 and 12 out of the 14 variables that measure soil properties. This implies that differences in observed plot characteristics between trial and non-trial parcels partly capture differences in soil properties and hence that the predictions also only partially correct for the effect due to parcel selection.

Finally, the adjustment for skills and other socio-economic characteristics is done by replacing the averages of these variables for the community-selected farmers by the ones of the representative farmers. In all cases, Fig. 1 incudes the 90% confidence interval of each prediction, and Supplementary Table S13 online provides the value of the changes in prediction due to each adjustment and the p-value of its significance (in a two-sided test).

### Calculation of the returns of the input packages

The value-cost ratio presented in Table 1 is obtained by dividing the value of the additional yield by the cost of the input package used in the treatment plot but not in the control plot. Profits are calculated by subtracting the cost of the input package from the value of the additional yield. To obtain the cost of each input, we multiply the quantity of the input used in the trial plot by the price per kg at which the input was purchased. We then sum this across all inputs to obtain the cost of the input package. To obtain the value of the additional yield, we multiply the difference in production between the treated parcel and the control one by the price per kg of the crop, using the average price at which the crop was sold, obtained from the farmer survey data.

Labour costs were not included in the calculation of additional costs because labour effort is notably difficult to quantify and its valuation can vary between households. For the calculation before adjustments, labour costs will only affect the value-cost ratio if it differs between control and treatment plots. Practices (such as weeding, harrowing and gapping) were harmonized between plots so that labour costs for those tasks don’t differ. However, the application of the inputs and the time to harvest additional production would generate additional labour costs in treatment plots. Hence the value-cost provided can be interpreted as an upper bound. For the calculation after adjustments, further assumptions would be needed in order to put a value on the differences in labour costs between plots. As such assumptions could be hard to defend, we instead report costs and profits without labour costs, while being explicit about their exclusion.

### Discussion of the choice of specification

In the main specification used for Fig. 1, all variables can directly contribute to the predictions of yield increments. This satisfies the purpose of predicting variations in yield while being agnostic about how these changes are triggered. A possible alternative approach is to estimate the physical-biological yield function based on agronomic variables and then predict variations in agronomic variables as a function of management and socio-economic variables. Such a model would assume that human capital variables only affect yields through changes in agronomic variables. If this specification is correct then one should find that, when predicting yield increments with agronomic variables, the addition of management and socio-economic variables do not increase the predictive power. Supplementary Table S16 online sheds light on this question by presenting the adjusted R^2^ in the predictions of yields and yield increments with different sets of variables. Compared with a model that only includes the agronomic variables, the results show that the inclusion of management variables adds between 4 and 9 percentage points to the explanatory power of the regression, which is a substantial improvement. The inclusion of socio-economic characteristics adds 4–5 percentage points to the explanatory power. Hence socio-economic characteristics and/or the quality of management adds additional elements of information not captured by agronomic characteristics. We can therefore reject a specification that assumes that management and socio-economic characteristics would only affect yield increments through agronomic characteristics.

### Review of recently published papers

To provide evidence that the sources of discrepancies highlighted in this paper are common in the on-farm trial literature, and to complement insights from the references cited in the introduction, we reviewed articles published in 2018 in two top field journals that frequently publish results from on-farm trials: *Field Crops Research* and *Agriculture, Ecosystems and Environment*. We also reviewed articles published in 2018 *PNAS*, *Nature* and *Nature Plants* but did not identify any articles reporting yield results from on-farm trials Among the 88 papers that report on agronomic trials, we kept those that satisfy the following criteria: (1) being in a low or middle income country, (2) reporting yield results for annual crops, (3) being implemented on farmers’ fields with at least some farmer involvement, (4) not having a complex design with multiple treatments and replications on same plot (which are more akin to on-station trials); and (5) not being a paper of which the results from trials were described in more detail in previous papers. The majority of papers that were not further considered are on-station trials, which by definition are researcher managed and not of interest to the arguments in this paper.

In 2018 there are 16 articles that fit these criteria, including^44^, which reports on the agronomic findings of the soybean trials used for the current paper. Supplementary Table S1 online summarizes information for those 16 articles^44,60–74^ relating to the 5 possible sources of discrepancies, and documents that these are indeed common practices in recently published research. Column 3 shows that random selection of farmers is the exception, and also shows that many papers don’t specify selection criteria (discrepancy 4). The later is even more the case for the selection of plots (discrepancy 3). When papers describe the non-random selection criteria, it typically involves selection by extension agents, or it is based on visibility and farmers interests in trial participation. Such selection criteria are likely to generate a sample of farmers that are not representative of other farmers in the area.

For all agronomic trials, researchers provided (as expected) the inputs and technical guidelines regarding the specific innovation that was tested. However, column 5 and 6 document that, in the vast majority of cases, researchers also provide complementary inputs and technical assistance on practices that were not being tested. This confirms that management and effort are likely to differ substantially between trial plots and the typical plot of a representative farmer (discrepancy 2). Less than half of the reviewed papers account for these additional inputs and effort through (partial) profit or cost–benefit calculations.

Column 7 documents that it is common to omit certain plots from the analysis, with the indicated reasons varying between crop failures, abnormal harvest values and management mistakes (discrepancy 1). Certain plots were excluded in almost half of the cases, and it is further unclear for some of the others whether all plots were taken into account. None of the papers specifies whether any adjustment for plant density was done, so that it is difficult to evaluate the frequency of this practice.

In the case of multi-season trials, it is often the case that not all farmers continue after the first season (column 9), but none of the reviewed papers accounts for this selection.

Finally, column 10 indicates the recommendations formulated at the end of the papers often are targeted to extension services or farmers themselves, confirming that trials with different levels of researcher involvement are being used to make recommendations for farmer practices without further testing in farmer-managed trials.

### Ethics review

The research obtained IRB approval both from the IRB at JPAL-Europe at PSE and from the Maseno University Ethics Review Committee in Kenya. All research was performed in accordance with relevant guidelines and regulations, and informed consent was obtained from all participants.

## Supplementary information

Supplementary information

## Data Availability

All data and computer code that support the findings of this study are available at: https://openicpsr.org/openicpsr/project/120430/version/V1/view.
