# Supplementary material for: Reconciling yield gains in agronomic trials with returns under African smallholder conditions
Source: Sci Rep. 2020 Aug 31;10:14286. doi: 10.1038/s41598-020-71155-y (PMC7459313; doi:10.1038/s41598-020-71155-y)
Supplement: Supplementary file 1 — Supplementary information [file 41598_2020_71155_MOESM1_ESM.pdf]

Supplementary Information for

Reconciling yield gains in agronomic trials with returns under African smallholder conditions

Rachid Laajaj, Karen Macours, Cargele Masso, Moses Thuita and Bernard Vanlauwe

This PDF file includes:

Tables S1 to S16

Other supplementary materials for this manuscript include the following:

Data files and programs for analysis are posted here:
https://openicpsr.org/openicpsr/project/120430/version/V1/view

Table S1: Review of recently published studies based on on-farm trials

			Selection		Researcher provides:		Yield calculations		If multi-season:	Use
Ref	Country	Type of innovation tested	How are farmers selected?	How are plots selected?	Inputs other than those tested	Technical assistance on practices other than those tested	Accounts for increased inputs/effort	Plots with failed crops excluded	Accounting for farmers that stop the trial	Recommen-dation for:
	(1)	(2)	(3)	(4)	(5)	(6)	(7)	(8)	(9)	(10)
60	Nepal	practices	random	?	yes	no	partial profit	no (?)	-	extension/ farmers
61	Malawi	crop varieties	non-random	?	yes	yes	no	yes	-	extension/ farmers
62	Nepal	practices	?	?	yes	yes	profit	no (?)	NA	extension/ farmers
63	Lao PDR	practices	non-random	?	yes	yes	partial profit	no	no	farmers
64	South Africa	fertilizers	?	?	yes	yes	no	yes	NA	research
65	Thailand	practices	non-random	?	?	no	benefit-cost	no	no	extension/ farmers
66	Philippines	crop varieties	non-random	?	yes	yes	no	yes	-	research
67	Southern Africa	crop varieties + practices	non-random	?	no	no	no	yes	-	extension/ farmers
68	Ethiopia	practices	non-random	?	yes	yes	no	no	-	none
69	Ghana	fertilizers	non-random	non-random	yes	yes	no	no	-	extension
70	Uganda	crop varieties	?	?	yes	yes	no	yes	-	extension
71	10 SS African countries	crop varieties + fertilizers	?	?	no	yes	benefit-cost compared to rule-of-thumb	?	no	research?
72	Rwanda	fertilizers	?	?	yes	yes	no	no	NA	research/ extension
73	Ethiopia	fertilizers	non-random	non-random	yes	yes	no	yes	no	farmers
74	Malawi	fertilizers	non-random	?	yes	yes	no	yes	no	extension
44	Kenya	fertilizers	random + non-random	non-random	yes	yes	benefit-cost compared to rule-of-thumb	no	no	research/ extension

Note: "?" Indicates that paper does not specify. In column 3 and 4, "non-random" implies that paper describes a particular selection mechanism that is non-random (e.g. selection by extension agents, based on visibility, etc). In column 7, "profit" refers to calculations subtracting the cost of all inputs from the value of increased yields, "partial profit" refers to profit calculations that account for some but not all inputs, "benefit-cost" refers to calculations of the ratio of yield benefits versus input costs, and "benefit-cost compared to rule-of-thumb" implies that ratio is compared with a benchmark value (higher than 1). Column 9 "-" implies that the trial was only conducted one season, "NA" implies all farmers continued in all seasons, and "no" implies certain farmers stopped after the first season but selection is not accounted for.

Table S2A: Inputs by plots used in Maize and Soya trials

Maize Plots	Inputs	Equivalent quantity per acre	Timing
Plot 1	FARMER'S LOCAL SEED	10 kg	Planting
Plot 2	FARMER'S LOCAL SEED	10 kg	Planting
	PHYMYX Organic Fertilizer	250 kg	Planting
	MAVUNO PLANTING	158 kg	Planting
	MAVUNO TOP DRESSING	54 kg	Top dressing
Plot 3	Maize seed: DH 04 / KSTP 94	10 kg	Planting
Plot 4	Maize seed: DH 04 / KSTP 94	10 kg	Planting
	PHYMYX Organic Fertilizer	250 kg	Planting
	MAVUNO PLANTING	158 kg	Planting
	MAVUNO TOP DRESSING	54 kg	Top dressing
Plot 5	Maize seed: IR	10 kg	Planting
Plot 6	Maize seed: IR	10 kg	Planting
	PHYMYX Organic Fertilizer	250 kg	Planting
	MAVUNO PLANTING	158 kg	Planting
	MAVUNO TOP DRESSING	54 kg	Top dressing

KSTP94 seeds was used in half of the trials, and DH04 in the other half

Table S2B: Inputs by plots used in Maize and Soya trials

Soya Plots	Inputs	Equivalent quantity per acre	Timing
Plot 1	Soybean Seed : SB 19	16 kg	Planting
Plot 2	Soybean Seed : SB 19	16 kg	Planting
	Biofix/Legumefix	10g per kg of seed	Planting
Plot 3	Soybean Seed : SB 19	16 kg	Planting
	MINJINGU	92 kg	Planting
Plot 4	Soybean Seed : SB 19	16 kg	Planting
	Biofix/Legumefix	10g per kg of seed	Planting
	MINJINGU	92 kg	Planting
Plot 5	Soybean Seed : SB 19	40 kg	Planting
	SYMPAL	119 kg	Planting
Plot 6	Soybean Seed : SB 19	40 kg	Planting
	Biofix/Legumefix	10g per kg of seed	Planting
	SYMPAL	119 kg	Planting

All inputs are used at planting. Legumefix was used in half of the trials, and Biofix in the other half.

Table S3: Comparisons of characteristics of management, plots and farmers in the trials and outside the trials

VARIABLES	AVERAGE IN TRIAL PLOTS	DIFFERENCE TRIAL -OTHER PLOTS	DIFFERENCE COMMUNITY - RANDOMLY SELECTED
**MANAGEMENT**			
**OBSERVED EFFORT, WEED AND STRIGA [TRIAL VS SURROUNDING]**			
Quality of management	1.29	0.085***	0.040*
(index)	[0.01]	[0.007]	[0.021]
Absence of weed	-0.73	0.092***	0.015
(index)	[0.01]	[0.002]	[0.011]
Absence of striga	-0.41	0.029***	0.00096
(index)	[0.00]	[0.003]	[0.0087]
**PRACTICES [SELF DECLARED BY FARMERS]**			
Harrowed at least 2 times	0.49	0.29***	0.068
(dummy)	[0.02]	[0.02]	[0.042]
Did at least 2 weedings	0.78	0.32***	0.068*
(dummy)	[0.02]	[0.02]	[0.038]
Did gapping or thinning	0.61	0.11***	0.0071
(dummy)	[0.02]	[0.02]	[0.044]
**PLOT CHARACTERISTICS**			
**SOIL PROPERTIES [FROM SOIL SAMPLING IN TRIAL PLOTS ONLY]**			
pH (soil acidity)	5.45		0.044
	[0.04]		[0.081]
Electrical Conductivity	87.18		3.35
(µS/cm)	[2.45]		[4.91]
Calcium (cmol+/kg)	4.54		0.078
	[0.16]		[0.32]
Magnesium (cmol+/kg)	1.78		-0.061
	[0.07]		[0.13]
Potassium (cmol+/kg)	0.45		0.015
	[0.02]		[0.047]
Sodium (cmol+/kg)	0.06		-0.00029
	[0.00]		[0.0023]
Exchangeable Acidity	0.24		0.013
(cmol+/kg)	[0.02]		[0.033]
Zinc (ppm)	3.85		0.21
	[0.18]		[0.36]
Copper (ppm)	4.01		-0.11
	[0.16]		[0.32]
Manganese (ppm)	323.29		-9.62
	[8.65]		[17.3]
Iron (ppm)	82.23		3.58
	[1.91]		[3.81]
Organic Carbon (%)	1.44		0.022
	[0.03]		[0.050]
Total Nitrogen (%)	0.11		0.0020
	[0.00]		[0.0038]
Phosphorus, Mehlich3	10.75		-1.47
(ppm)	[1.77]		[3.54]

VARIABLES	AVERAGE IN TRIAL PLOTS	DIFERENCE TRIAL - OTHER PLOTS	DIFFERENCE COMMUNITY - RANDOMLY SELECTED
**PRESENCE OF STRIGA [FROM TRIAL PLOT VERIFICATION PHASE]**			
Striga seen at identification	0.09		0.20*
(scale 0-3, standardized)	[0.06]		[0.12]
Plot had striga in prior season	0.13		0.081
(scale 0-3, standardized)	[0.06]		[0.11]
Nb of years with striga in plot	0.1		0.065
(standardized)	[0.06]		[0.11]
**BASELINE SURVEY CHARACTERISTICS OF THE PLOTS**			
Plot quality (farmer's	2.93	-0.08*	0.16**
assessment - scale 1-5)	[0.04]	[0.04]	[0.080]
Plot inclination (farmer's	1.47	-0.05	0.077
assessment - scale 1-3)	[0.03]	[0.03]	[0.058]
Plot with striga (dummy)	0.07	-1.94***	-0.058
	[0.06]	[0.06]	[0.12]
Plot area (ha)	0.69	0.10**	0.10
	[0.04]	[0.04]	[0.071]
Plot had maize in prior season	0.76	0.29***	0.040
(dummy)	[0.02]	[0.03]	[0.047]
Plot had legume or fallow	0.13	-0.05**	-0.0038
(dummy)	[0.02]	[0.02]	[0.037]
Distance to plot (minutes)	4.94	-6.71***	-0.36
	[0.38]	[1.38]	[0.76]
Nb of fertilizers used in prior	0.55	0.12***	0.25***
season	[0.04]	[0.04]	[0.086]
Applied soil conservation	0.15	-0.02	0.044
(dummy)	[0.02]	[0.02]	[0.039]
Applied fertilizer (dummy)	0.39	0.08***	0.13**
	[0.03]	[0.03]	[0.053]
Applied manure (dummy)	0.31	0.05*	0.064
	[0.03]	[0.03]	[0.050]
**SKILLS AND SOCIOECONOMIC CHARACTERISTICS OF FARMERS**			
**SKILLS OF THE PERSON RESPONSIBLE OF THE TRIALS**			
Cognitive skills (index)	0.07		0.43***
	[0.05]		[0.10]
Non-cognitive skills(index)	0.08		0.38***
	[0.05]		[0.10]
Agricultural knowledge skills	0.05		0.39***
(index)	[0.05]		[0.095]
**OTHER SOCIOECONOMIC CHARACTERISTICS**			
Wealth index	0.09		0.48***
	[0.06]		[0.11]
Respondent is the hh head	0.67		0.043
(dummy)	[0.03]		[0.052]
Respondent is female	0.57		-0.092*
(dummy)	[0.03]		[0.054]

Standard errors of means in brackets. * significant at 10%; ** significant at 5%; *** significant at 1% (two-sided tests). The third column presents the difference between the value of the variable in the trial and its value for the other plots of the same farmer (when applicable and available in the data). The last column presents the difference between the average value of the community selected farmers and the one of the randomly selected farmers. Standard errors are reported in parentheses below the differences. Because soil samples were only collected for trial plots, we are unable to compare the soil properties of trial plots with other plots. Similarly, plot observations at the plot identification stage are only available for trial plots, explaining why the "Difference trial-other plot" column is empty for these variables. See Methods section for definitions and units of all variables.

Table S4: Yield Increment Predictions using Observation of Management in Trials

VARIABLES	maize yield increment	soya yield increment
Quality of management		461.6***
		(157.4)
Absence of observed weed	2,467***	363.1
	(595.0)	(532.7)
Absence of observed Striga	-1,499**	
	(667.6)	
Observations	564	264
R-squared	0.070	0.089
Adjusted R-squared	0.0671	0.0825

Yield increment measured in kg/ha. Standard errors clustered at the household level in parentheses. *** p<0.01, ** p<0.05, * p<0.1 (Two-sided t-test). To deal with multicollinearity, starting from the full set of covariates for each outcome, a stepwise selection of variables with backward elimination was used with the adjusted R-squared as information criteria. As a result, only the subset of covariates maximizing the adjusted R-squared is retained for each regression. Subplot-pair binary variables are included in all regressions.

Table S5: Adjusted R-squared when predicting yield Increments

SET OF VARIABLES	Maize yield increment	Soya yield increment	Source	Observed in other plots?
Observed Management	0.067	0.083	Observation of agronomists	yes (surrounding of trials)
Self-declared management	0.019	0.010	Household survey	yes (asked for all plots)
Soil properties	0.072	0.063	Soil Sampling	No
Plot Characteristics (before trial)	0.026	0.075	Household survey	yes (asked for all plots)
Farmer's Skills	0.004	0.001	Psychometric measures & tests	Not Applicable
Other Socio-Economic Characteristics	0.019	0.010	Survey	Not Applicable
**Combining all Variables**	**0.192**	**0.260**		

For conciseness this table only presents adjusted R2. For the full regressions see Table S4, S5-S10. As in Table S4, in order to deal with multicolinearity, the subset of variables that has the highest adjusted R-squared was retained for each regression.

Table S6: Yield Increment Predictions using Management Self-declared by Farmers

VARIABLES	maize yield increment	soya yield increment
Did at least 2 weedings	-242.9	-78.19
	(154.6)	(98.07)
Did gapping or thinning	205.5	113.1
	(150.1)	(91.30)
Observations	540	260
R-squared	0.022	0.017
Adjusted R-squared	0.0187	0.00950

Yield increment measured in kg/ha. Standard errors clustered at the household level in parentheses. *** p<0.01, ** p<0.05, * p<0.1 (Two-sided t-test). To deal with multicollinearity, starting from the full set of covariates for each outcome, a stepwise selection of variables with backward elimination was used with the adjusted R-squared as information criteria. As a result, only the subset of covariates maximizing the adjusted R-squared is retained for each regression. Subplot-pair binary variables are included in all regressions.

Table S7: Yield Increment Predictions using Soil Properties from Soil Sampling

VARIABLES	maize yield increment	soya yield increment
PH (soil acidity)	-357.8**	
	(149.7)	
Electrical Conductivity	-3.058**	
	(1.377)	
Calcium	65.64**	28.16
	(31.06)	(22.71)
Magnesium		-46.22
		(35.43)
Potassium		316.0**
		(130.0)
Exchangeable Acidity	-184.8	123.8
	(200.4)	(113.9)
Zinc		-17.40*
		(9.947)
Copper	-33.80	
	(21.71)	
Manganese	0.904**	
	(0.406)	
Iron		0.861
		(1.051)
Organic Carbon	-543.9*	-89.36
	(307.5)	(76.29)
Total Nitrogen	3,915	
	(3,924)	
Phosphorus (Mehlich-3)		-3.805***
		(1.207)
Observations	537	254
R-squared	0.086	0.093
Adjusted R-squared	0.0717	0.0633

Yield increment measured in kg/ha. Standard errors clustered at the household level in parentheses. *** p<0.01, ** p<0.05, * p<0.1 (Two-sided t-test). To deal with multicollinearity, starting from the full set of covariates for each outcome, a stepwise selection of variables with backward elimination was used with the adjusted R-squared as information criteria. As a result, only the subset of covariates maximizing the adjusted R-squared is retained for each regression. Subplot-pair binary variables are included in all regressions.

Table S8: Yield Increment Predictions using Plot Characteristics Self-declared in Baseline Survey

VARIABLES	maize yield increment	soya yield increment
Plot quality (farmer's assessment)	-34.97	-59.16
	(58.44)	(45.02)
Plot inclination		-79.12
		(53.16)
Plot area (ha)	83.74	
	(80.36)	
Plot had maize in prior season	-173.7	207.5***
	(184.5)	(77.68)
Plot had legume or fallow	-282.1	
	(203.8)	
Distance to plot (minutes)	-6.068	6.801
	(8.256)	(5.313)
Number of fertilizers used in prior season		-35.01
		(36.14)
Applied fertilizer (dummy)	-70.59	
	(106.2)	
Applied manure (dummy)	-168.5	
	(102.1)	
Observations	555	254
R-squared	0.038	0.093
Adjusted R-squared	0.0257	0.0751

Yield increment measured in kg/ha. Standard errors clustered at the household level in parentheses. *** p<0.01, ** p<0.05, * p<0.1 (Two-sided t-test). To deal with multicollinearity, starting from the full set of covariates for each outcome, a stepwise selection of variables with backward elimination was used with the adjusted R-squared as information criteria. As a result, only the subset of covariates maximizing the adjusted R-squared is retained for each regression. Subplot-pair binary variables are included in all regressions.

Table S9: Yield Increment Predictions using Farmers Skills (from tests and psychometric measures)

VARIABLES	maize yield increment	soya yield increment
Cognitive skills	49.11	-26.49
	(48.01)	(37.10)
Agricultural knowledge skills	-64.62	44.02
	(55.27)	(36.22)
Observations	561	260
R-squared	0.007	0.009
Adjusted R-squared	0.00369	0.000867

Yield increment measured in kg/ha. Standard errors clustered at the household level in parentheses. *** p<0.01, ** p<0.05, * p<0.1 (Two-sided t-test). To deal with multicollinearity, starting from the full set of covariates for each outcome, a stepwise selection of variables with backward elimination was used with the adjusted R-squared as information criteria. As a result, only the subset of covariates maximizing the adjusted R-squared is retained for each regression. Subplot-pair binary variables are included in all regressions.

Table S10: Yield Increment Predictions using other Socio-Economic Variables (from survey)

VARIABLES	maize yield increment	soya yield increment
Wealth index	-42.13	22.39
	(51.19)	(28.50)
Respondent is the head of hh	117.0	-70.99
	(125.1)	(59.17)
Respondent is female	-85.52	
	(108.5)	
Observations	558	254
R-squared	0.025	0.018
Adjusted R-squared	0.0193	0.0104

Yield increment measured in kg/ha. Standard errors clustered at the household level in parentheses. *** p<0.01, ** p<0.05, * p<0.1 (Two-sided t-test). To deal with multicollinearity, starting from the full set of covariates for each outcome, a stepwise selection of variables with backward elimination was used with the adjusted R-squared as information criteria. As a result, only the subset of covariates maximizing the adjusted R-squared is retained for each regression. Subplot-pair binary variables are included in all regressions.

Table S11: Yield Increment Predictions when Including all Variables

VARIABLES	maize yield increment	soya yield increment	maize yield increment	soya yield increment
Quality of management	158.5	539.1***	158.5	539.1***
	(321.4)	(182.8)	(281.9)	(169.6)
Absence of observed weed	2,056**	600.0	2,056**	600.0
	(943.0)	(514.7)	(836.4)	(489.2)
Absence of observed Striga	-1,957***		-1,957**	
	(730.7)		(738.6)	
Did at least 2 weedings	-302.8		-302.8	
	(187.5)		(180.8)	
PH (soil acidity)	-314.4**		-314.4**	
	(144.4)		(144.5)	
Electrical Conductivity	-1.904		-1.904	
	(1.592)		(1.613)	
Calcium	85.91***	46.17**	85.91**	46.17**
	(31.31)	(19.21)	(32.95)	(20.37)
Potassium	-161.5	142.7	-161.5	142.7
	(136.7)	(114.3)	(145.8)	(131.6)
Sodium		2,389		2,389
		(1,789)		(1,973)
Exchangeable Acidity		84.57		84.57
		(110.5)		(102.8)
Zinc	-18.17	-19.71**	-18.17	-19.71*
	(24.72)	(9.599)	(21.24)	(10.07)
Copper	-19.42	-21.18*	-19.42	-21.18
	(24.07)	(11.83)	(24.00)	(12.80)
Manganese	1.167***	0.342	1.167***	0.342
	(0.433)	(0.298)	(0.346)	(0.297)
Organic Carbon	-646.3**	-118.9	-646.3**	-118.9
	(312.6)	(103.7)	(291.4)	(89.30)
Total Nitrogen	4,779		4,779	
	(4,138)		(3,945)	
Phosphorus (Mehlich-3)		-1.655		-1.655
		(1.063)		(1.132)
Striga seen at identification	-35.15		-35.15	
	(42.93)		(42.24)	
Plot had Striga in prior season	-53.51		-53.51	
	(47.84)		(48.48)	
Plot quality (farmer's assessment)	65.07	-64.73	65.07	-64.73
	(71.50)	(43.86)	(75.93)	(43.10)
Plot Inclination	-47.71	-157.7***	-47.71	-157.7***
	(90.13)	(59.86)	(82.14)	(58.48)
Plot with Striga		-64.72**		-64.72**

		(29.82)		(29.91)
Plot area (ha)	63.77	-65.09	63.77	-65.09
	(101.2)	(46.17)	(107.9)	(50.54)
Plot had maize in prior season	-307.7	394.5**	-307.7	394.5**
	(203.3)	(159.8)	(201.6)	(169.9)
Plot had legume or fallow	-381.3	291.6*	-381.3	291.6*
	(239.0)	(164.3)	(268.2)	(158.3)
Distance to plot (minutes)		13.27**		13.27**
		(5.954)		(6.205)
Applied soil conservation (dummy)		100.6		100.6
		(89.91)		(98.71)
Applied fertilizer (dummy)		-172.6**		-172.6**
		(74.38)		(82.33)
Applied manure (dummy)	-162.3		-162.3	
	(110.8)		(96.87)	
non-cognitive skills		-27.31		-27.31
		(31.72)		(34.88)
Agricultural knowledge skills		63.72		63.72
		(40.68)		(42.33)
community selected (dummy)		91.89		91.89
		(67.98)		(73.18)
Respondent is the head of hh	201.0	-159.7**	201.0	-159.7*
	(131.7)	(69.59)	(133.4)	(79.63)
Respondent is female	-113.0	-89.38	-113.0	-89.38
	(115.1)	(64.26)	(123.3)	(63.30)
Level of clustering of Standar Error	household	household	village	village
Observations	489	236	489	236
R-squared	0.231	0.345	0.231	0.345
Adjusted R-squared	0.192	0.260	0.192	0.260

Yield increment measured in kg/ha. Standard errors clustered at the household level in parentheses in the first two columns and at the village level in the last two columns. *** p<0.01, ** p<0.05, * p<0.1 (Two-sided t-test). To deal with multicollinearity, starting from the full set of covariates for each outcome, a stepwise selection of variables with backward elimination was used with the adjusted R-squared as information criteria. As a result, only the subset of covariates maximizing the adjusted R-squared is retained for each regression. Subplot-pair binary variables are included in all regressions.

Table S12: The effects of the calculation method on yield and yield increment

	MAIZE		SOYA	
**Yield Calculation Method:**	**Yield in Control Subplot**	**Yield Increment**	**Yield in Control Subplot**	**Yield Increment**
Population adjusted<br>Missing if no yield	1525	1103	1652	815
No population adjustment<br>Missing if no yield	1205<br>*[0.000]*	1182<br>*[0.172]*	903<br>*[0.000]*	367<br>*[0.000]*
No population adjustment<br>Zero if no yield	1075<br>*[0.000]*	1094<br>*[0.389]*	801<br>*[0.001]*	326<br>*[0.029]*

Yield and yield increment (kg/ha) measured using the different calculation methods described in the main text. The yield is the one in the control subplot, without input, and the yield increment compares the treated subplots T2 for maize and T6 for soya (similarly to Figure 1). The change between the first and the second line reflects the removal of the plant density adjustment. The change between the second and third line reflects the incorporation of the 0-yields in the yield calculation. In Brackets: p-value of whether it is significantly different from the calculation method above (two-sided tests). As the two-sided tests in the last line can only compare observations that are non-missing for both variables, they exclude observations that are missing due to yield failures in all plots of the same parcel.

Table S13: Changes due to the Adjustments and their Significance Level

Adjustment	maize yield increment	soya yield increment
**Calculations**	32<br>[0.262]	-341<br>[0.000]
**Management**	-77<br>[0.418]	-159<br>[0.001]
**Soil**	148<br>[0.008]	-133<br>[0.066]
**Socio-Economic**	-16<br>[0.119]	-27<br>[0.581]

The table presents the changes that occurred in each prediction with each adjustment as it appears in Figure 1. Below the value of the change are the p-value of whether this change is significantly different from 0.

Table S14: Yield increments for all treatment-control pairs in the maize, soya and intercrop trials

	MONOCROP								INTERCROP				
	MAIZE YIELD INCREMENT			SOYA YIELD INCREMENT					SOYA YIELD INCREMENT				
Treatment-control plot pairs compared for yield increment:	T2_C	T4-T3	T6-T5	T6-C	T4-C	T5-C	T3-C	T2-C	T6-C	T4-C	T5-C	T3-C	T2-C
Agronomic (Ag calculations)	1,065	828	805	692	197	509	128	175	340	272	443	175	135
**Agronomic (A)**	1,097	872	766	351	256	180	116	142	22	7	21	27	-1
A + Management adjustment	1,019	795	689	192	97	71	7	33	26	10	-9	-3	-32
A + Soil & Management adjustments	1,167	943	837	60	-35	-33	-97	-71	62	46	14	21	-8
**All adj (A+Management+Soil+Other)**	1,151	927	821	33	-62	-28	-92	-66	60	44	19	26	-3
Break even yield increment	274	274	274	55	43	28	16	26	77	65	49	36	26
Initial Value-Cost Ratio	3.89	3.02	2.94	12.49	4.57	18.43	8.21	6.67	4.41	4.19	9.10	4.82	5.12
Value-Cost Ratio after adjustments	4.20	3.38	2.99	0.59	-1.44	-1.00	-5.90	-2.52	0.77	0.68	0.40	0.71	-0.10

Yield increment measured in kg/ha. The first line shows the agronomic calculation that corrects for plant population, the second line applies no adjustment, and the following lines apply the adjustments based on predictions of yields under different conditions, as described in the text. The break even yield increment provides the yield increment that would allow the added value to be exactly equal to the cost of the added input between control and treated plot. The Value-Cost ratio divides the value of yield increment by the incremental value of input, and it is calculated separately using the agronomic calculation and the calculation after all adjustments.

Table S15: Yield in the trials, and definition of treatments

S15.A All farmers together

Maize yield by plot (kg/ha)

Plot 1	1525 [58]	Plot 2	2593 [68]
Plot 3	1444 [61]	Plot 4	2349 [67]
Plot 5	1292 [57]	Plot 6	2070 [69]

Soya yield by plot(kg/ha)

Plot 1	1652 [90]	Plot 2	1860 [103]
Plot 3	1848 [95]	Plot 4	1945 [105]
Plot 5	2152 [103]	Plot 6	2452 [121]

S15.B Only Community-Selected farmers

Maize yield by plot (kg/ha)

Plot 1	1543 [80]	Plot 2	2647 [88]
Plot 3	1469 [80]	Plot 4	2350 [93]
Plot 5	1276 [85]	Plot 6	2105 [103]

Soya yield by plot(kg/ha)

Plot 1	1758 [142]	Plot 2	1905 [145]
Plot 3	1993 [150]	Plot 4	2002 [134]
Plot 5	2117 [135]	Plot 6	2435 [170]

S15.C Only Randomly-Selected farmers

Maize yield by plot (kg/ha)

Plot 1	1505 [84]	Plot 2	2531 [104]
Plot 3	1416 [93]	Plot 4	2348 [98]
Plot 5	1310 [75]	Plot 6	2032 [91]

Soya yield by plot(kg/ha)

Plot 1	1546 [111]	Plot 2	1814 [147]
Plot 3	1704 [116]	Plot 4	1888 [162]
Plot 5	2186 [156]	Plot 6	2469 [173]

Yields observed in each plot (in kg/ha) averaged over the 3 seasons, using the agronomic calculation. Yield increment used in the calculations is the difference between a treatment plot, where the full input package was used, and its control plot. For maize the treatment plots are plots 2, 4 and 6 (all with Mavuno (10N:26P:10K: 5S: 14CaO +micronutrients), and Phymix (a vermicompost with total N (0.88%), organic C (7.31%), available P (0.39%), Ca (0.29%), Mg (0.1%), K (0.22%), and a pH that is approximately neutral (6.7%), compared to the control plot 1, 3 and 5 respectively, which has the same seed but no fertilizer. In the case of soya, plots 4 and 6 are the treatment plots (where a soybean rhizobia inoculant was tested together with Minjingu hyper phosphate (0-30-0+38CaO) or Sympal (0:23:15+10CaO+4S+1MgO+0.1Zn), and plot 1 is the control plot, also with same seed but no input. The full description of inputs tested in the plots appears in Table S2 and in the "Treatment structure and application" subsection in Methods. Standard errors in brackets.

Table S16: Adjusted R-squared when predicting yield Increments in order to test model specification

SET OF VARIABLES	maize yield increment	soya yield increment
Agronomic: Soil Properties & Plot Characteristics (before trial)	0.109	0.149
Agronomic + Farmers Skills & Other Socio-Economic Characteristics	0.148	0.189
Agronomic + Management (observed and self-declared)	0.150	0.236

For conciseness this table only presents adjusted R2. It follows the specification of Table S11 but varies the set of regressors included (specified in the first column). As in Table 11, in order to deal with multicolinearity, the subset of variables that has the highest adjusted R2 was retained for each regression.
